# *iDOM*: Statistical analysis of dissolved organic matter characterized by high‐resolution mass spectrometry

**DOI:** 10.1002/mlf2.70002

**Published:** 2025-04-14

**Authors:** Fanfan Meng, Ang Hu, Kyoung‐Soon Jang, Jianjun Wang

**Affiliations:** ^1^ State Key Laboratory of Lake and Watershed Science for Water Security, Nanjing Institute of Geography and Limnology, Chinese Academy of Sciences Nanjing China; ^2^ University of Chinese Academy of Sciences Beijing China; ^3^ Bio‐Chemical Analysis Team, Korea Basic Science Institute Cheongju South Korea

**Keywords:** dissolved organic matter, ecological interpretation, FT‐ICR MS, R package, statistical analysis

## Abstract

Dissolved organic matter (DOM) contains thousands of molecules and is key for biogeochemical cycles in aquatic and terrestrial ecosystems by interacting with microbes. Over the last decade, the study of DOM has been advanced and accelerated with the developments of instrumental and statistical approaches. However, it is still challenging in statistical analyses, data visualization, and theoretical interpretations largely due to the complexity of molecular composition and underlying ecological mechanisms. In this study, we developed an R package *iDOM* with functions for the basic and advanced statistical analyses and the visualization of DOM derived from Fourier transform ion cyclotron resonance mass spectrometer (FT‐ICR MS). The package could handle various data types of DOM, including molecular compositional data, molecular traits, and uncharacterized molecules (i.e., dark matter). It could integrate explanatory data, such as environmental and microbial data, to explore the relationships between DOM and abiotic or biotic drivers. To illustrate its use, we presented case studies with an example dataset of DOM and microbial communities under experimental warming. We included case studies of basic functions for the calculation of molecular traits, the assignment of molecular classes, and the compositional analyses of chemical diversity and dissimilarity. We further showed the case studies with advanced functions to quantify DOM assembly processes, assess the effects of dark matter on molecular interactions, analyze the ecological networks between DOM and microbes, and explore their response to warming. The source code and example dataset of *iDOM* are publicly available on https://github.com/jianjunwang/iDOM. We expect that *iDOM* will serve as a comprehensive pipeline for DOM statistical analyses and bridge the gap between chemical characterization and ecological interpretation in a theoretical framework.

## Impact statement

Dissolved organic matter (DOM) is a key component of Earth's ecosystems, playing a vital role in nutrient cycling and interactions with microbial communities. However, studying DOM presents significant statistical challenges due to its complex molecular composition and the difficulty of connecting its composition to ecological processes. To address these challenges, we developed *iDOM*, a new R package designed to streamline the analysis, visualization, and interpretation of DOM data. By integrating DOM data with environmental and microbial datasets, *iDOM* enables researchers to better understand how DOM responds to global changes, such as climate warming. This tool bridges disciplines of chemistry and ecology, providing novel insights into the DOM's role in ecosystem functioning.

## INTRODUCTION

Dissolved organic matter (DOM) is a large and complex mixture of thousands of molecules that play crucial roles in biogeochemical cycles^1^. The high heterogeneity of DOM composition has previously presented a great challenge for our understanding of their reactivity, persistence, and ecological significance^2^, ^3^. However, DOM studies have made substantial progress recently due to the advancements in high‐resolution mass spectrometry and statistical approaches. For instance, Fourier transform ion cyclotron resonance mass spectrometry (FT‐ICR MS) was first applied to characterize the molecular composition of humic and fulvic acids from the Suwannee River^4^. In the last decade, FT‐ICR MS has been increasingly used to examine DOM in natural environments such as lakes, rivers, oceans, and soils, and it has great potential for future studies^5^. The number of DOM studies using FT‐ICR MS reached 189 in 2023, reflecting a nearly 530% increase since 2014, and the percentage of these studies among all DOM studies rose from 2.50% to 8.32% (Figure S1). Compared to conventional bulk analysis methods like absorbance and fluorescence spectroscopy, FT‐ICR MS provides more detailed information on the elemental composition and molecular characteristics of DOM across global environments^6^. There were also advances in statistical approaches and relevant graphical tools, including the modified aromaticity index (AI_mod_)^7^ and the van Krevelen diagram^8^, which are widely used to assess DOM structural features and to visualize molecular composition based on H/C and O/C ratios, respectively.

New statistical and modeling approaches have been developed to substantially enhance our understanding of DOM and address key questions related to its assembly processes, environmental responses, and interactions with microbial communities. These advancements have shifted DOM research from traditional compositional analyses to a more functional perspective on DOM assemblages. One such advancement is the application of functional diversity to DOM through approaches like the community‐weighted mean (CWM), which simplifies complex mass spectral data by focusing on compositional‐level DOM traits^5^, ^8^. Furthermore, due to various traits among individual molecules, it is challenging to predict how DOM assemblages are assembled and how they respond to global change. These challenges are now being overcome by newly developed methods, such as the DOM partitioning framework (DOM_Part_)^9^, the indicator of compositional‐level environmental response (iCER)^10^, and the standardized Shannon index of DOM‐microbe associations (*H*_2_′)^11^. For instance, the DOM_Part_ categorizes DOM composition by considering molecular traits such as reactivity and activity and explains how variations in DOM fractions are shaped by both deterministic and stochastic processes. By applying ecological null models, it could estimate the relative contributions of these processes to the assembly of each fraction^9^. The indicator iCER could assess the overall environmental response of DOM assemblages, such as their thermal response, by aggregating the diverse responses of molecules that are positively or negatively associated with warming^10^, ^12^. The *H*_2_′ index could quantify the degree of specialization between DOM and microbes by analyzing their bipartite networks, which are based on resource‐consumer relationships^11^. Notably, in previous analyses, a large proportion of DOM compounds remain uncharacterized and neglected, often referred to as chemical “dark matter”. The new indicator of dark matter effects (iDME), developed based on ecological networks, could assess how dark matter influences molecular interactions within DOM assemblages under global change^13^. So far, there are a few open‐source R packages^14^ and pipelines^15^ developed for analyzing and visualizing FT‐ICR MS data. However, no software is available to integrate the advanced analyses mentioned above to understand the DOM composition within the ecological context.

**Figure 1:**
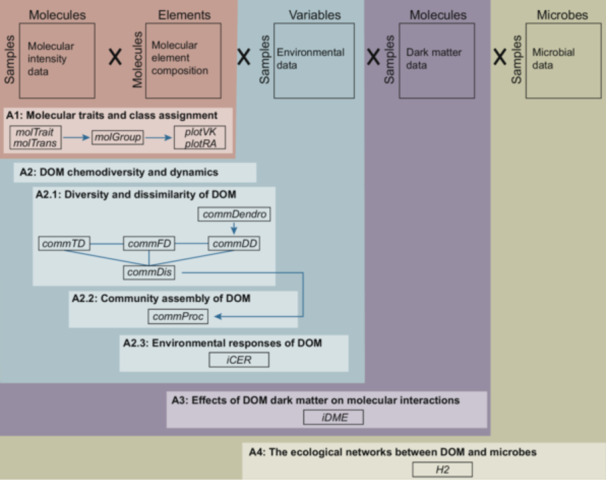
Conceptual scheme of R package *iDOM*. The *iDOM* package includes five example datasets: molecular intensity data, molecular element composition, environmental data, dark matter data, and microbial data. Using these datasets, the package addresses four main aims: (1) calculating molecular traits and assigning molecular classes; (2) analyzing DOM chemodiversity and dynamics, including (2.1) quantifying molecular diversity and dissimilarity, (2.2) explaining the assembly processes of DOM, and (2.3) assessing the environmental responses of DOM; (3) examining the effects of DOM dark matter on molecular interactions; and (4) quantifying DOM‐microbe associations through ecological networks.

Here, we develop an R package *iDOM* to bridge the current gap in advanced statistical analyses of FT‐ICR MS data (Figure 1). *iDOM* is a multifunctional tool that integrates both basic and advanced analytical functions for DOM research. The package's key features include basic analyses, such as the calculation of molecular traits, the assignment of molecular classes, and the calculation of chemical diversity and dissimilarity. It also offers advanced analyses to quantify the assembly processes of DOM composition^9^, the compositional‐level environmental responses of DOM^10^, the effects of dark matter^13^, and the specialization of DOM‐microbe associations^11^. Additionally, *iDOM* provides visualization functions, such as van Krevelen diagrams to visualize FT‐ICR MS data, and composition plots to show the relative abundances of different molecular classes. To illustrate its application, we applied the package to analyze an example dataset of DOM from a microcosm experiment investigating sediment DOM under experimental warming^10^.

## RESULTS AND DISCUSSION

### Calculation of molecular traits and assignment of molecular classes

The R package *iDOM* provides the *molTrait* and *molTrans* functions to calculate molecular properties and putative biochemical transformations, respectively, and the *molGroup* function to classify DOM molecules based on these traits. Specifically, the *molTrait* function calculates the chemical characteristics of molecules related to molecular weight, stoichiometry, chemical structure, and others (Tables 1 and S1). These traits include mass, the number of carbon atoms (C), Kendrick defect (kdefect_CH2_), O/C ratio, H/C ratio, N/C ratio, P/C ratio, S/C ratio, the modified aromaticity index (AI_mod_), double bond equivalent (DBE), DBE minus oxygen (DBE_O_), DBE minus AI (DBE_AI_), standard Gibbs Free Energy (GFE), nominal oxidation state of carbon (NOSC), and carbon use efficiency (Y_met_)^7^, ^16^, ^17^, ^18^, ^19^. Furthermore, the *molTrans* function estimates putative biochemical transformations for each molecule by comparing the mass differences between the molecule and others with a database of known transformations^20^.

The *molGroup* function classifies DOM composition into different groups based on various molecular traits and graphical methods, such as molecular properties, putative biochemical transformations, and van Krevelen diagrams^8^, ^20^. For instance, the assigned molecules can be classified into molecular formula groups based on their element composition (e.g., CHO, CHON, CHONS, CHONSP). Additionally, each molecule plotted on the van Krevelen diagram can be linked to specific natural biomolecules^8^. Molecules in different regions of the diagram are categorized into distinct classes, such as lipids, proteins, carbohydrates, lignin, and others^21^, ^22^. Recently, a new method was developed to classify molecules based on molecular reactivity, determined by H/C ratios, and activity, inferred from putative biochemical transformations^9^. This classification divides molecules into four fractions: labile‐active, recalcitrant‐active, labile‐inactive, and recalcitrant‐inactive.

**Figure 2:**
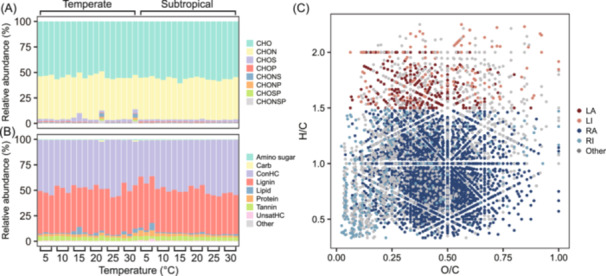
Molecular group composition and van Krevelen diagram. (A) Relative abundance of molecular groups based on element composition across experimental temperatures. (B) Relative abundance of molecular groups classified by H/C and O/C ratios across experimental temperatures. These groups include lipids (O/C = 0–0.3, H/C = 1.5–2.0), proteins (O/C = 0.3–0.55, H/C = 1.5–2.2), amino sugars (O/C = 0.55–0.67, H/C = 1.5–2.2), carbohydrates (Carb; O/C = 0.67–1.2, H/C = 1.5–2), unsaturated hydrocarbons (UnsatHC; O/C = 0–0.1, H/C = 0.7–1.5), lignin (O/C = 0.1–0.67, H/C = 0.7–1.5), tannin (O/C = 0.67–1.2, H/C = 0.5–1.5), and condensed aromatics (ConHC; O/C = 0–0.67, H/C = 0.2–0.7)^8^. (C) Molecular distributions in van Krevelen diagram. Different colors represent molecular groups classified by molecular trait dimensions of reactivity and activity: LA (labile‐active; H/C ≥ 1.5, transformations > 10), LI (labile‐inactive; H/C ≥ 1.5, transformations ≤ 1), RA (recalcitrant‐active; H/C < 1.5, transformations > 10), RI (recalcitrant‐inactive; H/C < 1.5, transformations ≤ 1), and others^9^.

For the example datasets, the *molGroup* function revealed that CHO and CHON formula groups consistently exhibited higher relative abundances than other groups across the temperature gradient from 5°C to 30°C (Figure 2A). Additionally, condensed aromatics (ConHC) and lignin consistently showed dominance throughout the temperature range (Figure 2B). The *molGroup* function also classified 4150 out of 5474 molecules into four fractions based on molecular reactivity and activity: labile‐active, recalcitrant‐active, labile‐inactive, and recalcitrant‐inactive. In both the labile and recalcitrant fractions, active molecules exhibited higher relative abundances than inactive molecules (Figure S2). Compared to traditional classifications based solely on molecular reactivity, such as an H/C ratio cutoff of 1.5, molecular activity offers new insights into the functional dynamics of molecules^9^, ^23^. Notably, in the van Krevelen diagram, highly active molecules are distributed across regions both above and below the H/C ratio threshold of 1.5 (Figure 2C), highlighting the limitations of such conventional criteria.

### Diversity and dissimilarity of DOM

The R package *iDOM* provides the *commTD*, *commFD*, and *commDD* functions to calculate within‐assemblage diversity and the *commDis* function to assess between‐assemblage differences in DOM composition (Figure 1). To apply diversity metrics, originally designed for ecological species, to molecular data, individual molecules are treated as species, with the relative intensities of their peaks representing species abundances. The *commTD* function calculates taxonomic diversity using common alpha‐diversity indices, including richness, abundance‐based metrics, and evenness. It computes molecular richness as the number of molecular formulas and calculates abundance‐based diversity metrics, such as Shannon and Gini‐Simpson (or Simpson's index), which integrate both molecular richness and relative intensities^24^, ^25^. Meanwhile, it measures evenness (e.g., Pielou's evenness), derived from molecular richness and the Shannon index, to reflect the equality of molecular intensity distributions. The *commFD* function calculates functional structure and diversity based on molecular traits, using approaches such as the CWM and Rao's quadratic entropy (RaoQ). The CWM simplifies complex mass spectral data into compositional‐level DOM traits, while RaoQ measures the average abundance‐weighted trait differences between pairs of molecules within an assemblage^26^. Greater trait differences among individual molecules result in higher quadratic entropy, indicating increased functional diversity^27^.

To further explore relationships among molecules, the *commDendro* function generates molecular dendrograms analogous to phylogenetic trees. These dendrograms are constructed based on molecular properties and putative biochemical transformations, including the molecular characteristics dendrogram (MCD), the transformation‐based dendrogram (TD), and the transformation‐weighted characteristics dendrogram (TWCD), to represent shared and divergent molecular traits among molecules^20^. Using these molecular dendrograms, the *commDD* function calculates dendrogram‐based diversity metrics, including dendrogram diversity (DD), mean pairwise distance (MPD), and mean nearest taxon distance (MNTD)^28^. DD quantifies the total branch length of a dendrogram occupied by a molecular assemblage, similar to Faith's Phylogenetic Diversity^29^. MPD measures the average dendrogram distance between molecules, while MNTD calculates the average dendrogram distance between nearest neighbors. For instance, molecular assemblages with higher DD values reflect a broader range of molecular properties in MCD, a more extensive biochemical transformation network in TD, or a combination of both in TWCD^20^.

As a complement to alpha diversity, the *commDis* function compares differences in DOM composition between samples by generating a dissimilarity matrix. The dissimilarity metrics include incidence‐based Jaccard, abundance‐based Bray‐Curtis, dendrogram‐based UniFrac, and others. Each dissimilarity matrix can be further analyzed using nonmetric multidimensional scaling (NMDS) or principal coordinate analysis (PCoA) to visually depict the relationships among samples.

**Figure 3:**
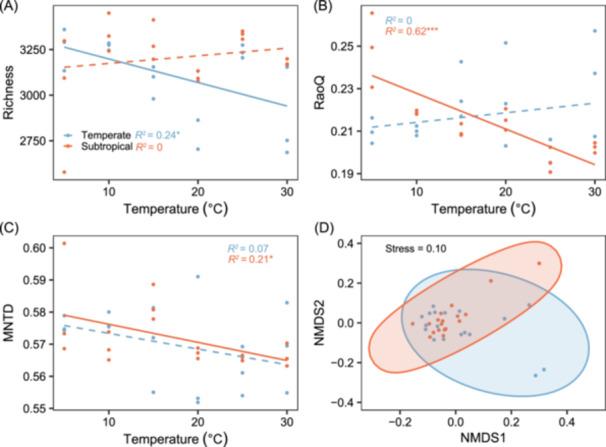
Relationships between DOM chemodiversity and experimental temperatures. (A–C) Molecular richness (A), Rao's quadratic entropy (RaoQ) (B) based on the modified aromaticity index (AI_mod_), and mean nearest taxon distance (MNTD) (C) derived from the molecular characteristics dendrogram (MCD) across experimental temperatures in temperate and subtropical regions. The relationships between these indices and experimental temperatures were tested using linear models. Solid lines indicate statistically significant models (*p* < 0.05), while dashed lines represent nonsignificant models (*p* > 0.05). Significance levels are indicated as follows: ***p < 0.001; *p < 0.05. (D) Compositional dissimilarity among samples visualized using NMDS based on Bray‐Curtis distance.

Based on the example datasets, the *commTD*, *commFD*, and *commDD* functions revealed that DOM molecular diversity exhibited distinct relationships with experimental temperature in temperate and subtropical regions. The molecular richness decreased significantly with rising temperature in the temperate region (*p* < 0.05), while no significant change was observed in the subtropical region (*p* > 0.05) (Figure 3A). Conversely, RaoQ and MNTD decreased significantly with rising temperature in the subtropical region (*p* < 0.05), but showed no significant change in the temperate region (*p* > 0.05) (Figure 3B,C). Further, the *commDis* function revealed that molecular composition was similar between the subtropical and temperate regions, as indicated by Permutational Multivariate Analysis of Variance (PERMANOVA, *p* > 0.05) (Figure 3D).

### Community assembly of DOM composition

Identifying how deterministic and stochastic processes influence changes in DOM assemblages is crucial for predicting their turnover and dynamics in response to global change^20^, ^30^. The *commProc* function quantifies the relative contributions of these processes to DOM assembly using dendrogram‐based *β*‐diversity null modeling^9^. Specifically, the function calculates the dendrogram‐based *β*‐nearest taxon index (*β*NTI) to measure tip‐level clustering or overdispersion within a molecular dendrogram, analogous to its use in phylogenetic trees for ecological communities^31^, ^32^. The *β*NTI is calculated by comparing the observed *β*‐mean nearest taxon distance (*β*MNTD_obs_) between pairs of DOM assemblages to null expectations (*β*MNTD_null_), generated by randomizing observed dendrogram associations^20^. The formula is as follows:

$$\beta \text{NTI}=\frac{{\beta \text{MNTD}}_{\text{obs}}-\bar{{\beta \text{MNTD}}_{\text{null}}}}{\text{sd}\left({\beta \text{MNTD}}_{\text{null}}\right)},$$

where *β*MNTD_obs_ represents the observed *β*MNTD, $\bar{{\beta \text{MNTD}}_{\text{null}}}\;$is the mean *β*MNTD for the null assemblages, and sd(*β*MNTD_null_) indicates the standard deviation of null values. If the comparison between two DOM assemblages significantly deviates from the null expectations (|*β*NTI| > 2), deterministic processes likely drive the observed patterns. Positive *β*NTI values (*β*NTI > 2) indicate divergent molecular composition resulting from variable selection, while negative *β*NTI values (*β*NTI < −2) suggest convergent molecular composition driven by homogeneous selection^9^. Conversely, if the comparison aligns with the null expectations (|*β*NTI| < 2), stochastic processes are likely responsible for the observed patterns^9^.

**Figure 4:**
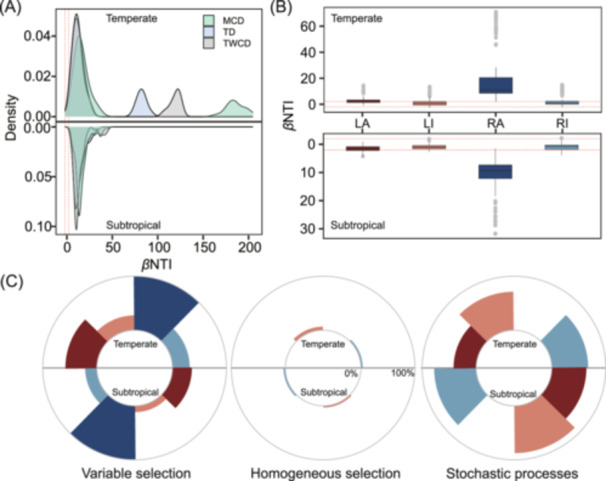
Community assembly of DOM composition. (A) Distribution of *β*‐nearest taxon index (*β*NTI) values derived from three molecular dendrograms: MCD, transformation‐based dendrogram (TD), and transformation‐weighted characteristics dendrogram (TWCD). (B) Boxplot of *β*NTI values of different DOM fractions based on the MCD. In the example datasets, 4150 out of 5474 molecules across 36 samples were classified into four fractions based on their reactivity and activity: LA, RA, RI, and LI. (C) Relative contributions of assembly processes, including variable selection, homogeneous selection, and stochastic processes, to the four DOM fractions. For the example datasets, the assembly processes of these four fractions were quantified using incidence‐based molecular data, that is, the presence or absence of molecular peaks rather than peak intensity data. This is because peak intensities could not contain the same ecological meaning as variations in microbial species abundances. The use of peak intensities from FT‐ICS MS is challenging for null modeling without additional information linking peak intensities to concentrations^9^.

For the example datasets, we used the *commProc* function to quantify the relative contributions of deterministic and stochastic processes to DOM assembly. The majority of *β*NTI values for MCD, TD, and TWCD exceeded 2, indicating that deterministic processes, particularly variable selection, predominantly governed molecular assembly (Figure 4A). To further explore the assembly mechanisms of different DOM fractions compared to whole DOM assemblages, we applied the *molGroup* function to classify DOM composition into four fractions based on molecular reactivity and activity (Figure 4B). In both subtropical and temperate regions, deterministic processes driven by variable selection dominated the assembly of labile and recalcitrant molecules in the active fractions, while stochastic processes are more important for the assembly of molecules within the inactive fractions. The homogeneous selection had minimal importance across all fractions in both regions (Figure 4C).

### Thermal responses of DOM composition

The diverse intrinsic traits of DOM molecules present a significant challenge in predicting their responses to climate change and, consequently, the impact on the global carbon cycle. The *iCER* function is designed to quantify the responses of DOM to environmental changes, such as warming, at both molecular and compositional levels^10^. The thermal responses of DOM assemblages can be quantified using a novel indicator, iCER, which is based on changes in molecular abundances along temperature gradients. The calculation of iCER involves two main steps. First, the *iCER* function determines the direction and magnitude of the molecule‐specific environmental response (MER) to temperature. MER quantifies the effect size of changes in the relative abundance of each molecule in response to temperature variations across different time, space, or treatments. Positive MER values are associated with molecules that accumulate as temperatures increase (warm‐accumulating molecules), while negative MER values are linked to molecules that deplete with elevated temperatures (warm‐depleting molecules). Second, the *iCER* function calculates the iCER indicator by taking the weighted average of the MERs within each DOM assemblage. This approach enables iCER to capture the overall response of a DOM assemblage to temperature changes, reflected in the balance between positive and negative MERs. A positive iCER value indicates that the DOM assemblage is dominated by warm‐accumulating molecules, while a negative value suggests the dominance of warm‐depleting molecules. An iCER value of zero suggests an equal representation of both groups. Since iCER depends on MER, it is essential to use statistically independent datasets: one for calculating MERs and the other for iCERs. This ensures that the relative abundances of individual molecules would not be reused, allowing iCER to be derived independently from MER values^10^.

**Figure 5:**
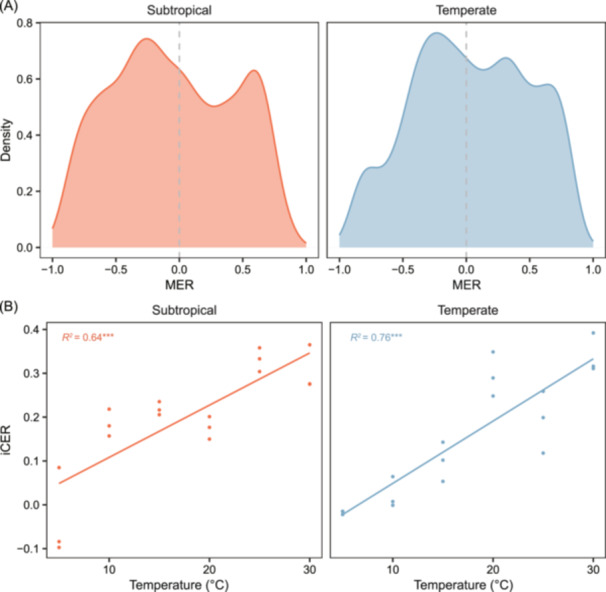
The molecule‐specific environmental response (MER) and the indicator of compositional‐level environmental response (iCER) of DOM. (A) Distribution of MERs for DOM molecules in subtropical and temperate regions. MERs were calculated as Spearman's correlation coefficients. (B) Relationships between iCERs and experimental temperatures in subtropical and temperate regions tested using linear models. iCERs were derived from molecules with statistically significant MERs (*p* < 0.05). Solid lines indicate statistically significant models (*p* < 0.05). ***p < 0.001.

We randomly partitioned the example datasets into two independent subsets for MER and iCER calculations using the commonly applied 80:20 ratio from species distribution modeling and machine learning^33^. Specifically, 80% of the samples (i.e., MER dataset) in each region were used to calculate the MERs of individual molecules, and the remaining 20% (i.e., iCER dataset) were then used to calculate the iCER for each sample based on the relative abundances of individual molecules and their corresponding MERs. In the MER dataset, we calculated MERs as Spearman's correlation coefficients (*ρ*) between the relative abundances of individual molecules and temperature. To avoid false correlations caused by low‐occurrence molecules, we retained only those molecules detected in at least 30% of the total samples for the MER calculation. Then, in the iCER dataset, we synthesized the MERs of a suite of molecules into an iCER for each sample. The iCER was calculated as:

$$\mathrm{iCER}=\frac{\sum (\;{\text{MER}}_{i}\times I_{i})}{\sum (I_{i})},$$

where MER_*i*_ represents the MER value of molecule *i*, and *I*_*i*_ indicates its relative abundance. This data partitioning and indicator calculations were randomly repeated 999 times, and the resulting MER and iCER values were averaged across all randomizations. For the example datasets, MER values ranged from −0.99 to 0.98 across both regions. The negative MER values had median values of −0.41 and −0.34, while the positive MER values had medians of 0.43 and 0.42 in the subtropical and temperate regions, respectively (Figure 5A). The iCER values exhibited consistent warming responses in the temperate and subtropical regions, with positive correlations with temperature and *R* ^*2*^ values of 0.76 and 0.64, respectively (Figure 5B). These results indicate that individual DOM molecules show varied thermal responses, while DOM assemblages consistently exhibit enhanced thermal responses under warmer conditions^10^.

### Effects of DOM dark matter on molecular interactions

**Figure 6:**
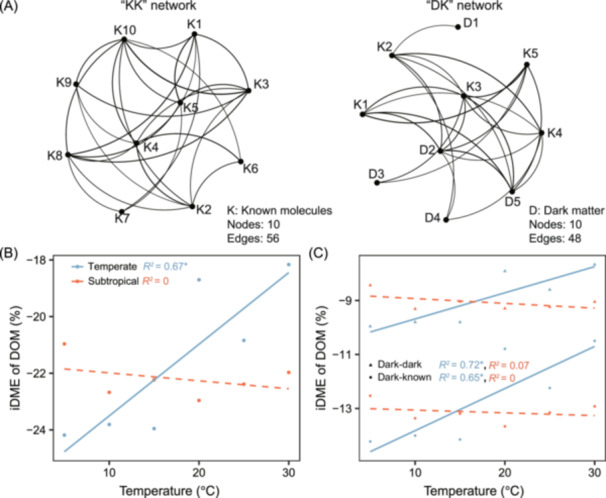
Molecular co‐occurrence networks and the indicator of dark matter effects (iDME). (A) Illustration of “KK” and “DK” molecular co‐occurrence networks with 10 nodes. The “KK” network includes only known molecules (“K”), while the “DK” network replaces half of the known molecules in the “KK” network with dark matter (“D”). The “KK” and “DK” networks were constructed using molecules present in more than 30% of the total samples, with interactions inferred through SparCC (Sparse Correlations for Compositional data)^34^. SparCC correlations below the threshold of |*ρ*| = 0.30 were excluded to remove uncorrelated or weakly correlated interactions. (B) Relationships between iDME and experimental temperatures in subtropical and temperate regions tested using linear models. For each sample in the example datasets, the iDME was calculated based on the “KK” and “DK” networks, each constructed using 400 nodes randomly sampled from its DOM composition, with the sampling repeated 100 times. The iDME values were averaged across different temperature levels. (C) Relationships between iDME partitions and experimental temperatures in subtropical and temperate regions tested using linear models. Dark–dark and Dark–known links correspond to the intra‐ and inter‐iDME partitions, respectively. Solid lines indicate statistically significant models (*p* < 0.05), while dashed lines represent nonsignificant models (*p* > 0.05). *p < 0.05.

DOM molecules detected by FT‐ICR MS can be assigned to identifiable molecular formulas, yet a large proportion still remains uncharacterized and is often referred to as chemical “dark matter”^13^. The interaction between chemical dark matter and assigned (i.e., known) molecules presents a significant challenge to fully understanding biogeochemical cycles. The *iDME* function calculates the iDME to quantify how dark matter influences molecular interactions within DOM assemblages by constructing co‐occurrence networks^13^. In each network, nodes represent individual molecules, and edges indicate the interactions between molecules. Specifically, two types of networks are constructed based on the presence or absence of dark matter: “KK” networks, which include only known molecules, and “DK” networks, which incorporate both dark matter and known molecules at a 1:1 ratio or according to the observed dark‐to‐known ratio in a DOM assemblage (Figure 6A). The “KK” and “DK” networks contain the same number of nodes, which are randomly subsampled from the entire DOM molecule pool and then replicated 100 times. The *iDME* function calculates the magnitude and direction of the iDME indicator by measuring the percentage change in the average value of a network metric *M*, such as degree centrality, between “KK” and “DK” networks.

$$\text{iDME }\left(\%\right)=\left(\frac{\bar{M_{\text{DK}}}}{\bar{M_{\text{KK}}}}-1\right)\times 100.$$

Degree refers to the number of edges connecting a focal node to other nodes within a network^35^. Molecules with a higher degree generally exhibit more interactions, indicating greater connectivity within an assemblage. Therefore, positive iDME values suggest that dark matter enhances network connectivity within a DOM assemblage, while negative values imply a reduction and an iDME of zero indicates a neutral effect. The iDME can be further divided into intra‐iDME and inter‐iDME to determine whether the effects of dark matter result from interactions between dark‐dark nodes or dark‐known nodes^13^.

For the example datasets, all iDME values calculated based on the network metric degree were negative and significantly different from zero, ranging from −24.2% to −18.2% in temperate regions and from −23.0% to −21.0% in subtropical regions. In temperate regions, iDME values increased significantly along the temperature gradient (*p* < 0.05), while subtropical regions showed nonsignificant trend (*p* > 0.05) (Figure 6B). These results suggest that DOM dark matter substantially reduced network connectivity along the temperature gradients in both regions, but its negative effects in temperate regions weakened as the temperature increased. Moreover, the partitioning of iDME showed that dark matter effects were primarily driven by changes in links between dark‐known nodes, followed by changes in links between dark–dark nodes in both temperate and subtropical regions (Figure 6C).

### The ecological networks between DOM and microbes

The fate of DOM is intimately linked to the metabolism of complex microbial communities, as microbes regulate the production and degradation of specific molecules^11^, ^36^. The associations between DOM molecules and microbial taxa can be quantified using DOM‐microbe bipartite networks, which are constructed based on resource‐consumer theory^37^. In these networks, individual DOM molecules are exclusively linked to microbial taxa that utilize them, while direct interactions among molecules or taxa are not considered. Based on resource–consumer relationships, negative network interactions likely reflect the degradation of larger molecules into smaller structures, while positive interactions may indicate the production of new molecules through either degradation or biosynthetic processes^11^.

The *H2* function calculates the *H*_2_′ index of DOM‐microbe bipartite networks to quantify specialization between DOM and microbes and standardizes it using null modeling to allow direct comparisons across samples^11^, ^37^. Specifically, an elevated *H*_2_′ indicates a high degree of specialization between DOM and microbes, with extreme cases where a single bacterial taxon might consume or produce only one specific DOM molecule. Conversely, a lower *H*_2_′ value suggests a more generalized bipartite network, where various DOM molecules are used by a wide range of bacterial taxa^11^.

**Figure 7:**
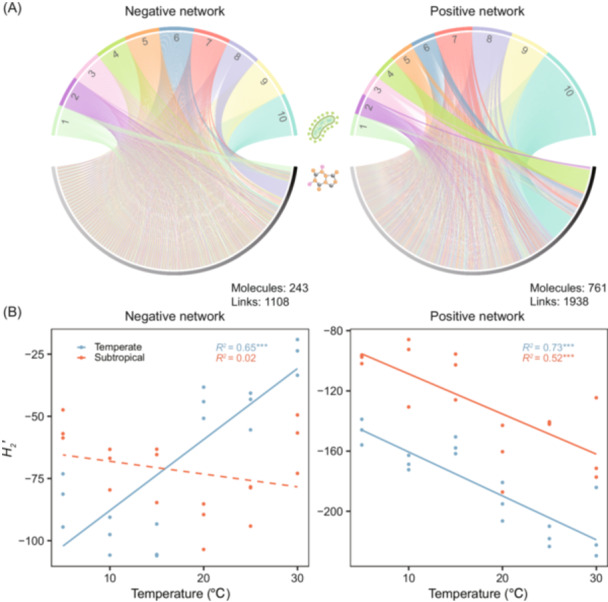
Specialization of DOM‐microbe bipartite networks. (A) Negative and positive bipartite networks between DOM molecules and bacterial genera. Upper arcs represent the top 10 bacterial genera, which showed more associations with molecules than other bacterial genera. Each genus is assigned a unique color and number. In the negative network, the genera are: 1, *Ramlibacter*; 2, *Anaeromyxobacter*; 3, *Caulobacter*; 4, *Magnetospirillum*; 5, *GOUTA19*; 6, *Treponema*; 7, *Symbiobacterium*; 8, *Kaistobacter*; 9, *Desulfurispora*; 10, *Rhodoplanes*. In the positive network, the genera are: 1, *Opitutus*; 2, *Sulfuricurvum*; 3, *Rhodoplanes*; 4, *Janthinobacterium*; 5, *Desulfurispora*; 6, *Azohydromonas*; 7, *Dehalobacterium*; 8, *Sphingomonas*; 9, *Paracoccus*; 10, *Kaistobacter*. Lower arcs represent DOM molecules, colored in shades from gray to black, with darker shades indicating molecules that have more associations with bacterial genera. Negative and positive networks were constructed based on DOM molecules and bacterial genera present in more than 30% of the total samples, using negative and positive SparCC correlation coefficients (*ρ* < −0.50 and *ρ* > 0.50, respectively). For each sample in the example datasets, separate negative and positive sub‐networks were constructed by selecting the DOM molecules and bacterial genera in each sample based on its DOM and bacterial compositions. In each subnetwork, SparCC *ρ* values were multiplied by 100,000, rounded to integers, and converted to absolute values. These values were then used to calculate standardized specialization indices (*H*_2_′) through null modeling using the shuffle.web algorithm. (B) Relationships between *H*_2_′ of negative and positive networks and experimental temperatures in subtropical and temperate regions, tested using linear models. Solid lines indicate statistically significant models (*p* < 0.05), while dashed line represents nonsignificant models (*p* > 0.05). ***p < 0.001.

We applied the *H2* function to the example datasets to examine how DOM‐microbe associations vary under experimental temperatures. In total, the negative and positive bipartite networks contained 1108 and 1938 interactions between DOM molecules and bacteria genera, respectively (Figure 7A). The standardized *H*_2_′ values were negative and significantly lower than expected by chance (*p* < 0.05), indicating that the interactions between DOM and bacteria were nonrandom. Experimental warming had contrasting effects on the *H*_2_′ of negative and positive networks across the two regions (Figure 7B). Specifically, for the positive networks, experimental warming significantly decreased *H*_2_′ in both temperate and subtropical regions (*p* < 0.05). For the negative networks, experimental warming significantly increased *H*_2_′ for the temperate region (*p* < 0.05), while no significant correlation was observed in the subtropical region. Experimental warming may thus enhance the recalcitrance of DOM in the temperate region by increasing production (i.e., less specialized positive networks) and reducing the decomposition of molecules (i.e., more specialized negative networks)^11^.

In summary, the package *iDOM* is a comprehensive set of functions developed to facilitate the chemical characterization and ecological interpretation of DOM based on high‐resolution mass spectrometry. *iDOM* enables us to perform chemical characterization, such as molecular trait calculation, molecular class assignment, and compositional analyses of chemical diversity and dissimilarity. Further, *iDOM* integrates concepts and tools from community ecology to facilitate the theoretical interpretation of community assembly of DOM, the thermal responses of DOM assemblages, the effects of dark matter on molecular interactions, and the DOM‐microbe associations. The *iDOM* is expected to promote standardized methodologies and reproducible research in DOM studies, and its extensibility makes it suitable for a wide range of applications across global environments.

## MATERIALS AND METHODS

### Description of functions in the R package

The *iDOM* package, developed in the R programming language, provides a variety of functions for the statistical analysis and graphical visualization of DOM, as summarized in Table 1. Key functions, such as *molGroup*, *commProc*, *iCER*, *iDME*, and *H2*, were specifically developed based on novel frameworks or indices reported in our previous literature^9^, ^10^, ^11^, ^13^. Other functions like *molTrait*, *molTrans*, *commTD*, *commFD*, *commDendro*, *commDD*, and *commDis* are necessary for supporting these primary functions. These additional functions rely on external packages, including *vegan* ^38^, *FD* ^39^, *picante* ^40^, *iCAMP* ^41^, and *ftmsRanalysis* ^14^, as well as R scripts from Danczak et al.^20^.

**Table 1:** The functions of R package “*iDOM*”.

Type	Function	Description
Molecular properties and class assignment	*molTrait*	To compute various molecular traits related to chemical characteristics
	*molTrans*	To estimate biochemical transformations for individual molecules
	*molGroup*	To classify DOM molecules into different groups based on various molecular traits and graphical methods
Diversity and dissimilarity of DOM	*commTD*	To calculate various taxonomic diversity and evenness metrics
	*commFD*	To calculate various functional diversity metrics
	*commDendro*	To generate relational metabolite dendrograms based on molecular traits and putative biochemical transformations
	*commDD*	To calculate various dendrogram‐based diversity metrics derived from metabolite dendrograms
	*commDis*	To calculate differences in DOM composition among samples
Community assembly of DOM	*commProc*	To evaluate the influence of deterministic and stochastic processes on DOM assemblages
Thermal responses of DOM	*iCER*	To quantify the responses of DOM to environmental changes, such as warming, at both the molecular and compositional levels
Effects of DOM dark matter	*iDME*	To assess the effects of dark matter on DOM assemblages
Associations between DOM and microbes	*H2*	To calculate the network‐level specialization in DOM‐microbe bipartite networks
Visualization	*plotVK*	To generate van Krevelen diagrams based on molecular O/C and H/C ratios
	*plotRA*	To visualize the relative abundances of different molecular groups

The *iDOM* package is designed with a modular structure, with its functions focused on four main aims (Figure 1). The first aim is to use molecular intensity and element composition data to calculate molecular traits with the *molTrait* and *molTrans* functions and classify molecular groups based on these traits using the *molGroup* function. The second aim is to integrate environmental variables to (1) describe the distribution of molecular diversity and dissimilarity using the *commTD*, *commFD*, *commDD*, and *commDis* functions; (2) explain the assembly processes of DOM composition with the *commProc* function; and (3) analyze the environmental responses of DOM through the *iCER* function. The third aim is to incorporate dark matter data to examine its effects on molecular interactions within DOM assemblages through ecological co‐occurrence networks with the *iDME* function. The fourth aim is to include microbial data to quantify DOM‐microbe associations based on bipartite networks using the *H2* function.

### Example datasets

The *iDOM* package provides example datasets of DOM and microbial communities under experimental warming. These datasets were generated from a laboratory microcosm experiment using sterilized Taihu Lake sediments as the organic carbon source, inoculated with distinct microbial communities from sediments of subtropical and temperate lakes in China^10^. The microcosms were incubated in the dark for 1 month at six temperature levels (5°C, 10°C, 15°C, 20°C, 25°C, and 30°C), with each temperature treatment replicated three times, resulting in a total of 36 samples across two climate zones^10^.

Five example datasets are included: molecular intensity data, molecular element composition, environmental data, dark matter data, and microbial data (Figure 1). The molecular intensity and element composition datasets provide the intensities of 5474 molecules and their corresponding element composition assignments across 36 samples. The environmental dataset contains variables such as experimental incubation temperatures, offering additional context for analyses under varying environmental conditions. The dark matter dataset comprises the intensities of 5779 unassigned molecules, offering insights into their effects on molecular interactions within DOM assemblages. The microbial dataset includes the relative abundances of 463 bacterial genera across 36 samples and can be used to investigate the interactions between DOM and bacteria.

### Data and computational requirements for the *iDOM* package

To effectively use the *iDOM* package, it is crucial to meet the minimum requirements of the mass spectral dataset, such as a sufficient sample size for robust statistical analyses. Additionally, the complete input dataset should comprise molecular intensity and element composition data, and supplementary data such as environmental variables, unassigned molecular data, and microbial data to explain the observed DOM composition. Depending on specific aims, users, however, can adjust the input data accordingly based on their dataset characteristics and research objectives. For large datasets, users are advised to use parallel computing methods available in other R packages or software. For instance, the calculation of co‐occurrence networks can be accelerated using *SpiecEasi* ^42^ or *fastspar* ^43^, which can reduce the overall computation time of the *iDME* and *H2* functions.

## AUTHOR CONTRIBUTIONS

**Fanfan Meng**: Formal analysis; writing—original draft. **Ang Hu**: Data curation; methodology; writing—review and editing. **Kyoung‐Soon Jang**: Data curation; methodology. **Jianjun Wang**: Data curation; methodology; project administration; supervision; writing—review and editing.

## ETHICS STATEMENT

The study in this article did not involve any trials on humans or animals.

## CONFLICT OF INTERESTS

The authors declare no conflict of interests.

## Supporting information

Supporting information.

## Data Availability

The *iDOM* open‐source software package is implemented in R and available for download via Github (https://github.com/jianjunwang/iDOM).
