# Supplementary material for: iDOM: Statistical analysis of dissolved organic matter characterized by high‐resolution mass spectrometry
Source: mLife. 2025 Apr 14;4(3):319–31. doi: 10.1002/mlf2.70002 (PMC12207904; doi:10.1002/mlf2.70002)
Supplement: Supplementary file 1 — Supporting information. [file MLF2-4-319-s001.docx]

**Supplementary Information:**

***iDOM*:** **Statistical analysis of dissolved organic matter characterized by high-resolution mass spectrometry**

Fanfan Meng^a, b,1^, Ang Hu^a,1^, Kyoung-Soon Jang^c^, Jianjun Wang^a,^*

^a^ Key Laboratory of Lake and Watershed Science for Water Security, Nanjing Institute of Geography and Limnology, Chinese Academy of Sciences, Nanjing 210008, China

^b^ University of Chinese Academy of Sciences, Beijing 100049, China

^c^ Bio-Chemical Analysis Team, Korea Basic Science Institute, Cheongju 28119, South Korea

^1^ These authors contributed equally to this work.

^*^ Corresponding author.

E-mail address: [jjwang@niglas.ac.cn](mailto:jjwang@niglas.ac.cn) (J. J. Wang).

Table S1. Descriptions of molecular traits

| **Group** | **Trait** | **Description** |
| --- | --- | --- |
| Molecular weight | Mass | The mass to charge ratio (m/z) |
| Molecular weight | C | The number of carbon |
| Molecular weight | kdefect_CH2_ | Kendrick Defect |
| Stoichiometry | O/C | O/C ratio |
| Stoichiometry | H/C | H/C ratio |
| Stoichiometry | N/C | N/C ratio |
| Stoichiometry | P/C | P/C ratio |
| Stoichiometry | N/P | N/P ratio |
| Stoichiometry | S/C | S/C ratio |
| Chemical structure | AI_mod_^a^ | The modified aromaticity index |
| Chemical structure | DBE^b^ | Double bond equivalence |
| Chemical structure | DBE_O_ | Double bond equivalence minus oxygen |
| Chemical structure | DBE_AI_ | Double bond equivalence minus aromaticity index |
| Oxidation state | GFE | Gibbs free energy |
| Oxidation state | NOSC^c^ | Nominal oxidation state of carbon |
| Carbon use efficiency | Y_met_ | Carbon use efficiency |

^a^$\mathrm{AI}_{\mathrm{mod}}=\frac{1+C-\frac{1}{2}O-S-\frac{1}{2}(N+P+H)}{C-\frac{1}{2}O-N-S-P}$;

^b^ $\mathrm{DBE}=1+\frac{1}{2}(2C-H+N+P)$;

^c^ $\mathrm{NOSC}=-(\frac{-Z+4C+H-3N-2O+5P-2S}{C})+4$, where *Z* is the net charge of the molecule and equals zero when formula lists contained only the neutral forms of the measured negatively ionized molecular formulae.


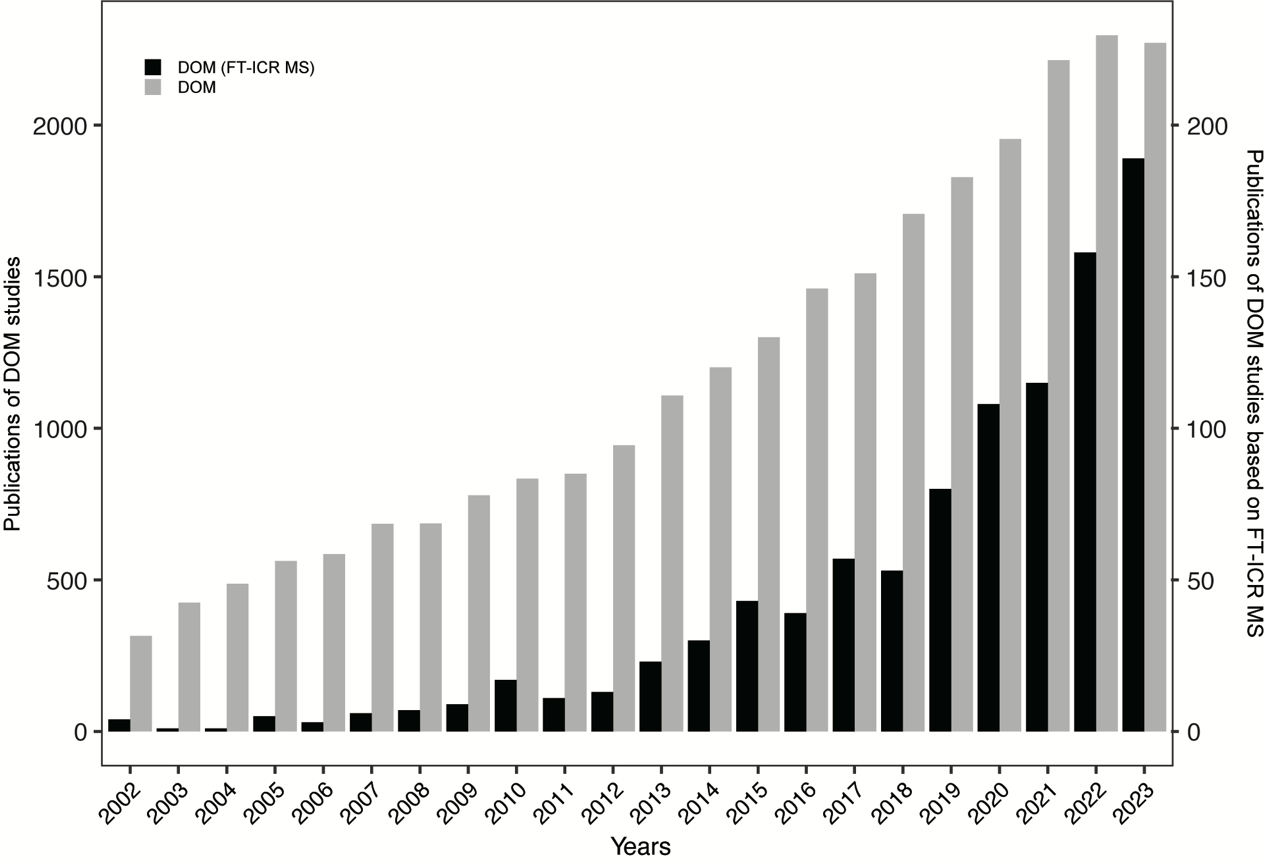


**Figure S1.** **The compilation of publications searched on the Web of Science**. We used the keyword (“Dissolved Organic Matter”) to search for all DOM studies, and the keywords (“Fourier Transform Ion Cyclotron Resonance Mass Spectrometry” OR “FT-ICR MS” OR “FTICR-MS”) AND (“Dissolved Organic Matter” OR “DOM”) to search for DOM studies using FT-ICR MS.


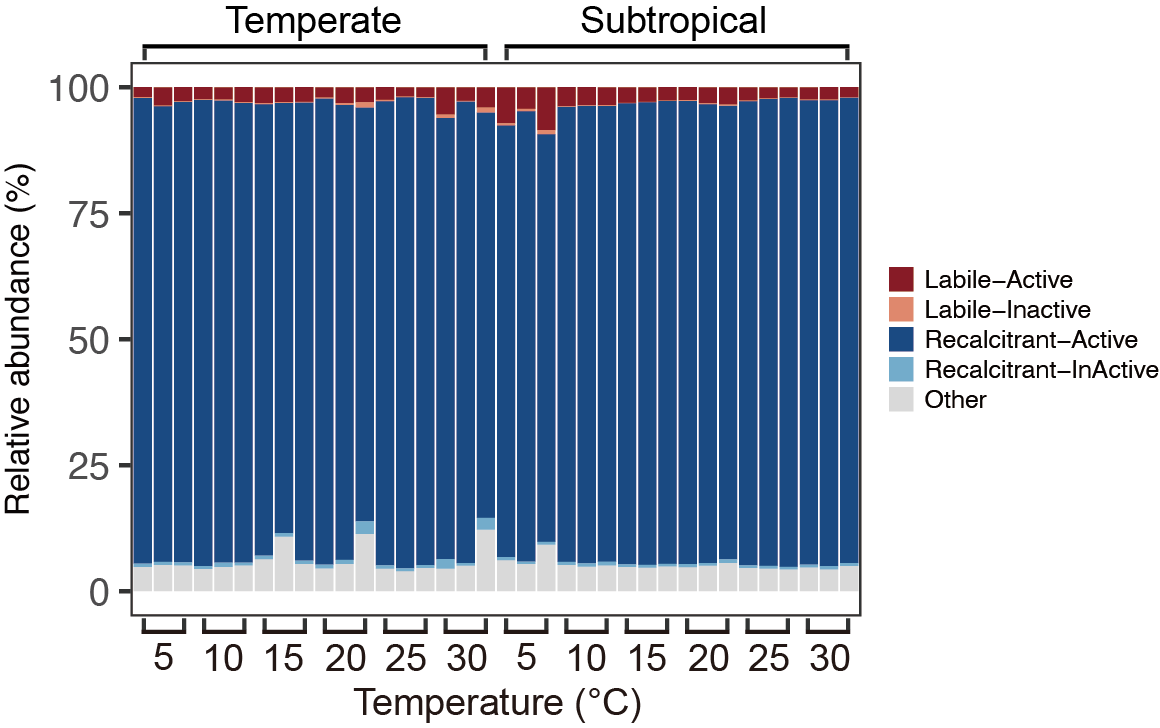


**Figure S2. The relative abundance of different molecular groups**. These groups were identified based on molecular H/C ratio and putative biochemical transformations: labile-active, recalcitrant-active, recalcitrant-inactive, and labile-inactive.
